# Identification of Quantitative Trait Loci Associated with Powdery Mildew Resistance in Spring Barley under Conditions of Southeastern Kazakhstan

**DOI:** 10.3390/plants12122375

**Published:** 2023-06-19

**Authors:** Yuliya Genievskaya, Alibek Zatybekov, Saule Abugalieva, Yerlan Turuspekov

**Affiliations:** 1 Laboratory of Molecular Genetics, Institute of Plant Biology and Biotechnology, Almaty 050040, Kazakhstan; y.genievskaya@ipbb.kz (Y.G.); a.zatybekov@ipbb.kz (A.Z.); s.abugalieva@ipbb.kz (S.A.); 2 Faculty of Biology and Biotechnology, Al-Farabi Kazakh National University, Almaty 050040, Kazakhstan

**Keywords:** *Hordeum vulgare* L., *Blumeria graminis* (DC.) f. sp. *hordei*, disease resistance, genome-wide association study, haplotypes, marker-assisted selection

## Abstract

Barley (*Hordeum vulgare* L.) is one of the most produced cereal crops in the world. It has traditionally been used for the production of animal feed and for malting, as well as for human consumption. However, its production is highly affected by biotic stress factors, particularly the fungal pathogen *Blumeria graminis* (DC.) f. sp. *hordei* (*Bgh*), which causes powdery mildew (PM). In this study, a collection of 406 barley accessions from the USA, Kazakhstan, Europe, and Africa were assessed for resistance to PM over a 3-year period in southeastern Kazakhstan. The collection was grown in the field in 2020, 2021, and 2022 and was genotyped using the 9K SNP Illumina chip. A genome-wide association study (GWAS) was conducted to identify the quantitative trait loci (QTLs) associated with PM resistance. As a result, seven QTLs for PM resistance were detected on chromosomes 4H, 5H, and 7H (FDR *p*-values < 0.05). Genetic positions of two QTLs were similar to those of PM resistance QTLs previously reported in the scientific literature, suggesting that the five remaining QTLs are novel putative genetic factors for the studied trait. Haplotype analysis for seven QTLs revealed three haplotypes which were associated with total PM resistance and one haplotype associated with the high PM severity in the barley collection. Identified QTLs and haplotypes associated with the PM resistance of barley may be used for further analysis, trait pyramiding, and marker-assisted selection.

## 1. Introduction

Barley (*Hordeum vulgare* L.) is one of the top cereal crops in the world [1], including in Kazakhstan [2]. However, in Kazakhstan, barley grain yield remains relatively low compared to leading barley-producing countries. In the 2022/2023 season, for example, barley grain yield in Kazakhstan was 1.5 t/ha, compared with 2.8 t/ha in Russia [3]. The difference can be explained by a number of factors, including poor tolerance/resistance of local cultivars to abiotic and biotic environmental factors. This includes a lack of genes providing durable resistance to fungal diseases. One of the most widespread fungal diseases affecting cereals is powdery mildew (PM). In barley, PM is caused by the obligate pathogen *Blumeria graminis* (DC.) f. sp. *hordei* (*Bgh*) [4]. The disease is prevalent in temperate regions of the Northern Hemisphere, including Kazakhstan [4]. Yield losses of 10–15% caused by PM in barley are common worldwide, but losses can reach 40% in temperate climate zones [5,6]. A relatively long vegetation period and a cool and humid climate are favorable for *Bgh* development. This is why PM is the most prevalent barley disease in Europe [7]. However, in recent years, fungal disease epidemics in barley have also been observed in southern and southeastern Kazakhstan [8], and expansive movement to other regions of the country has also reported [9].

PM and other fungal infections in barley fields have traditionally been controlled by fungicides and by the cultivation of resistant plants. Chemical protection provides conditions for the selection of fungicide-resistant strains [10]; however, the cultivation of resistant barley cultivars is the most economically and environmentally friendly method. Cultivating disease-resistant barley cultivars is the most effective strategy for controlling airborne diseases, such as PM [11]. Modern genetic resistance of barley to PM includes the durable race-specific resistance gene *mlo*, which has played a key role in plant resistance to PM in recent decades [12,13]. In barley, the gene *mlo* is located in the middle of the long arm of chromosome 4H, with more than 40 *mlo*-alleles identified [14,15]. However, despite the effectiveness of *mlo*, it does have several drawbacks. The gene produces a pleiotropic effect, so that necrotic areas on the leaf occur spontaneously; this leads to a reduction in photosynthesis, and therefore, to decreased kernel size and lower yields [13]. In addition, barley plants with the *mlo* resistance allele are susceptible to spot diseases, such as spot blotch [16] and Ramularia leaf spot [17]. The other important PM resistance gene that exhibits race-specific resistance to *Bgh* in barley is *Mla*, located on chromosome 1H [18]. Unfortunately, *Mla* also has drawbacks. Some *Mla* alleles require additional genes to express full resistance, such as *Rar1*-dependent *Mla10* [19].

Several other important PM resistance genes in barley have been described. These are *MlGa*, *Mlk*, *Mlnn*, and *Mlra* on chromosome 1H [20], *MlLa* and *MlMor* on chromosome 2H [7,21,22], *Mlg* and *MlBo* on chromosome 4H [7,23], *Mlj* on chromosome 5H [24], *Mlh* on chromosome 6H [7], *mlt* and *Mlf* on chromosome 7H [24], and many others. However, all these genes are race-specific, non-universal, and non-durable, and can therefore be easily overcome by new races of *Bgh* [25]. 

Despite a large number of identified PM resistance genes and alleles, new virulent *Bgh* pathotypes continue to be identified around the world. Despite the effectiveness of *mlo* in the last 50 years, *mlo*-virulent *Bgh* isolates have been reported in Europe, Japan, Australia, and China [14,26,27]. In Kazakhstan in 2015 and 2016, 107 isolates of *Bgh* were collected from seven populations of cultivated barley throughout the country and studied for virulence [28]. All isolates were virulent for *Mla8* and avirulent for *Mla9*, *Mla1* + *Mla2*, *Mla6* + *Mla14*, *Mla13* + *MlRu3*, *Mla7* + *MlNo3*, *Mla10* + *MlDu2*, *Mla13* + *MlRu3*, and *Mlo5* resistance genes [28]. Among 46 cultivars of Kazakhstani spring and winter barley which were evaluated for PM resistance in southeastern Kazakhstan in 2021–2022, 17 spring and 11 winter cultivars were found to be of the resistant (R) reaction type, while the remaining cultivars were moderately resistant (MR) or moderately susceptible (MS) [29]. These results mean that, in this host–pathogen race, we need to search for new resistance loci to pyramid them against PM and other barley diseases, while considering other agronomic traits.

Previously, restriction fragment length polymorphism (RFLP) [30], cleaved amplified polymorphic sequences (CAPS) [31], and simple sequence repeat (SSR) [32] markers were described for the selection of PM-resistant barley genotypes. However, in the past 15 years, genetic mapping of new quantitative trait loci (QTL) has developed rapidly. The traditional approach to genetic mapping of QTL is based on searching for associations between genotype and phenotype data from segregating populations resulting in bi-parental crosses. However, this process is time-consuming due to the development of bi-parental populations, and the genetic diversity of the resulting population is limited to parental genetic background. Currently, the most common and powerful method of QTL identification is the genome-wide association study (GWAS) based on linkage disequilibrium (LD) [33]. GWAS considers ancestral recombination events to identify significant associations between genotypic and phenotypic variations. Today, this method, along with traditional linkage mapping with bi-parental populations, is routinely used for the identification of QTLs in crops, including barley. During the last decade alone, GWAS in barley has been successfully used for the identification of QTLs for abiotic stress tolerance [34,35,36], adaptation traits [37,38,39], yield-related traits [40,41], grain components and quality traits [42,43,44], and resistance to diseases [45,46,47,48]. Novel PM resistance QTLs have been identified using GWAS in wheat [49,50], oat [51], rye [52], and, of course, barley [53,54,55,56]. However, in Kazakhstan, no GWAS of barley resistance to local *Bgh* pathotypes has yet been attempted. Therefore, the main goal of this research was to assess the resistance to PM of a diverse spring barley collection in southeastern Kazakhstan and to identify novel—and possibly unique—QTLs for local *Bgh* pathotypes.

## 2. Results

### 2.1. PM Resistance and Its Correlation with Agronomic Traits in the Studied Barley Collection

**Figure 1:**
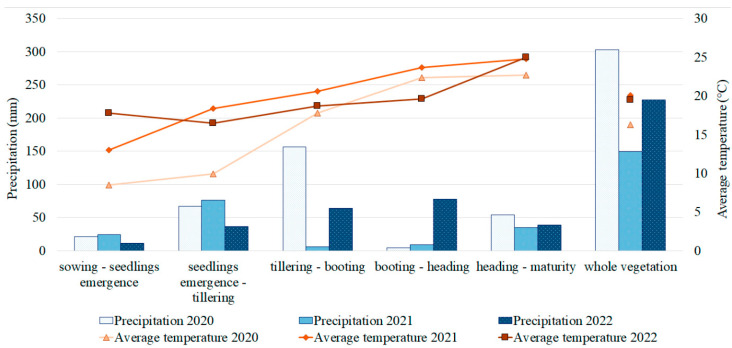
The weather conditions in the Kazakh Research Institute of Agriculture and Plant Growing (KRIAPG) fields over three years. Average temperature (°C) and precipitation (mm) during barley growth stages are indicated.

PM resistance was assessed in the Kazakh Research Institute of Agriculture and Plant Growing (KRIAPG) fields over three years (2020, 2021, and 2022). The weather conditions in 2021 were unfavorable for proper pathogen development (Figure 1), and no signs of PM were recorded. However, a phenotypic evaluation of PM in the barley collection in both 2020 and 2022 demonstrated the prevalence of resistant accessions with 0 points on the PM severity scale. In total, 351 and 397 accessions were resistant to PM in 2020 and 2022, respectively. The remaining accessions were evenly distributed among other PM resistance levels (Figure S1A,B). The Pearson correlation coefficient was used to evaluate the PM correlation with the three studied yield components: NKS, TKW, and YM2. The results of the correlation suggested that PM severity was negatively correlated with NKS under the conditions of 2020 (Figure S1C). However, none of the other correlation results, including mean values for the two years, suggested that PM severity negatively affected the yield components (Figure S1).

### 2.2. Populaton Structure, QTLs for Powdery Mildew PM Resistance, and Linkage Disequilibrium (LD) among Them

The population structure of the studied barley collection was previously described using 1648 SNPs and three methods: principal component analysis (PCA), neighbor-joining (NJ) tree, and Bayesian clustering [57]. Analysis results are presented in Figure S2. The PCA and NJ tree revealed the formation of the US cluster apart from the accessions from Kazakhstan, Europe, and Africa (Figure S2A,B). Accessions from Europe and Kazakhstan were clustered together on the PCA plot (Figure S2A), while, in the NJ tree, accessions from Europe were together with African ones (Figure S2B). The results of the STRUCTURE analysis revealed that *K* = 5 (number of subpopulations), implying the significant population structure in the studied barley collection (Figure S2C).

**Figure 2:**
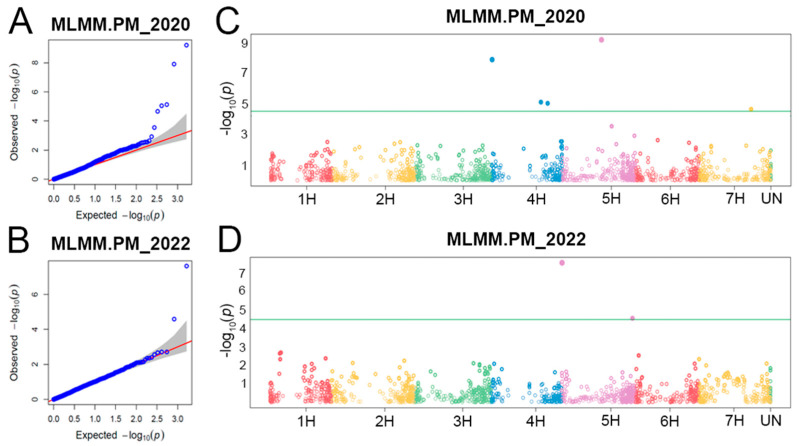
Genome-wide association study (GWAS) plots. Quantile–quantile (Q–Q) plots for powdery mildew (PM) resistance in (**A**) 2020 and (**B**) 2022. The diagonal red line represents normal distribution of the dots. Manhattan plots for PM resistance in (**C**) 2020 and (**D**) 2022. The green horizontal line represents the Bonferroni-adjusted significance threshold at −log10 (*p*) = 4.5. Different color of dots corresponds to seven chromosomes.

An MLMM model with K- and Q-matrices was successfully used for GWAS, demonstrating a good correlation between expected and observed *p*-values and outbreaks of significant associations (Figure 2A,B). In total, seven MTAs were detected on chromosomes 4H (3 MTAs), 5H (3 MTAs), and 7H (1 MTA) (Figure 2C,D, Table 1). Five MTAs were identified in 2020, and two MTAs were found in 2022. *p*-values of significant MTAs ranged from 2.6 × 10^−5^ (*Qhv_PM-5H.3*) to 6.2 × 10^−10^ (*Qhv_PM-5H.2*) with phenotypic variance explained (PVE-value) from 0.0% (*Qhv_PM-4H.2*, *Qhv_PM-4H.3*, and *Qhv_PM-5H.2*) to 85.3% (*Qhv_PM-4H.1*) (Table 1). Minor alleles of four MTAs negatively increased PM resistance, while minor alleles of the other three MTAs with positive effects increased PM severity in barley plants.

A comparative analysis of QTLs for PM resistance from the literature enabled the identification of candidate loci for two QTLs (*Qhv_PM-4H.1* and *Qhv_PM-5H.1*) (Table 1).

**Figure 3:**
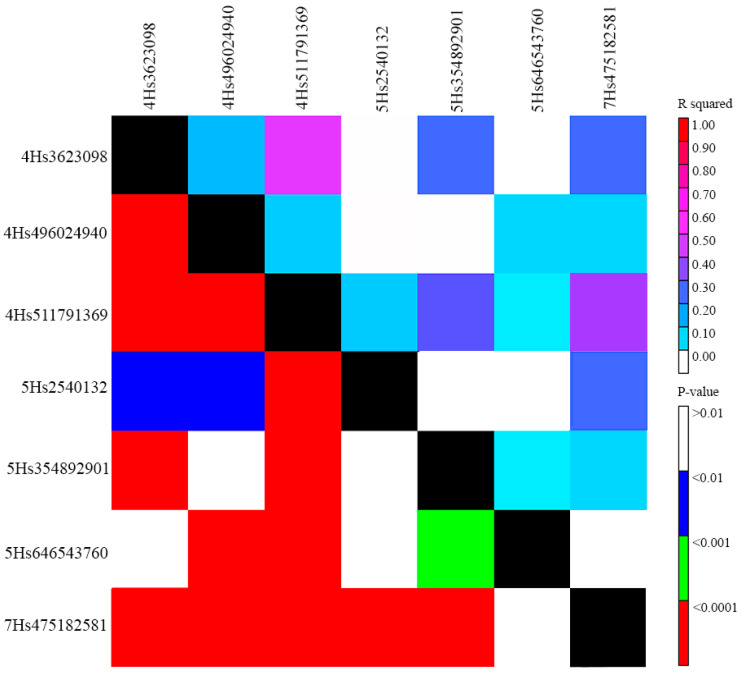
Linkage disequilibrium (LD) among SNPs associated with powdery mildew. The upper-right part of the plot is a heat map of *R^2^* values for each significant locus. Name of loci are their chromosome and position. The lower-left part is a heat map of *p*-values for each QTL. Color scales for *R^2^* and *p*-values are shown on the right.

LD analysis showed poor genetic linkage (*R^2^* < 0.2, *p* < 0.0001) in QTL (SNP) pairs *Qhv_PM-4H.1*/*Qhv_PM-4H.2* and *Qhv_PM-4H.2*/*Qhv_PM-4H.3* on chromosome 4H, but moderate linkage (*R^2^* = 0.52, *p* < 0.0001) between QTLs *Qhv_PM-4H.1* and *Qhv_PM-4H.3* on the same chromosome (Figure 3). However, the distance between these SNPs was large (508,168,271 bp, Table 1), and therefore, they were considered unlinked. On chromosome 5H, all three QTLs (SNPs) were unlinked (*R^2^* < 0.1, *p* < 0.05) (Figure 3). On chromosome 7H, there was only one QTL (Table 1). LD among QTLs (SNPs) on different chromosomes was not considered.

### 2.3. Effect of Haplotypes

**Figure 4:**
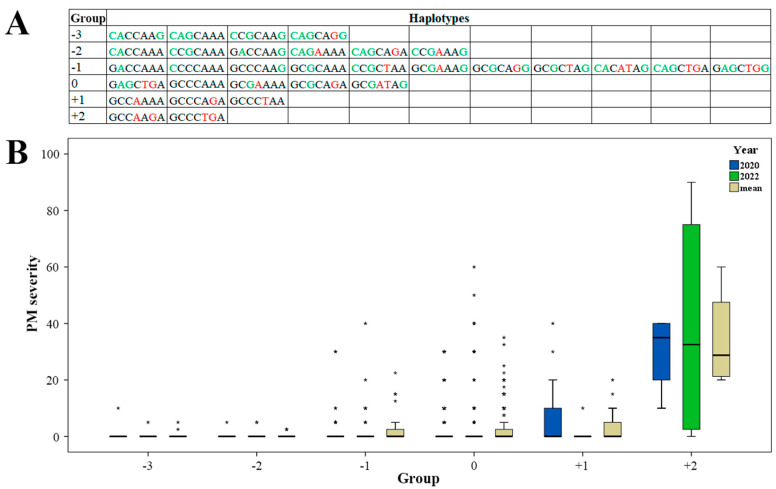
Effects of haplotype groups on powdery mildew (PM) resistance. (**A**) Distribution of haplotypes by groups according to their predicted effect (points) on powdery mildew (PM) resistance. (**B**) Boxplots showing the effect of each haplotype group on PM resistance in the studied barley population, with values for 2020, 2022, and the mean of the two years. *—outliers.

In order to find favorable combinations of alleles, haplotypes were formed based on the significant markers. Alleles in haplotypes were evaluated according to their effect from GWAS. Alleles with a negative effect on PM severity received −1 point, neutral alleles received 0 points, and alleles increasing PM severity received +1 point. The total effect of haplotype on PM severity was calculated as a sum of alleles’ points. Haplotypes with the same effects were grouped together. In total, 31 haplotypes distributed among six groups were found in the studied barley collection (Figure 4A).

The boxplot in Figure 4B illustrates PM severity in six haplotype groups measured in 2020 and 2022, and mean values. The lowest disease severity was observed in haplotypes from groups “−3” and “−2” with several outbreaks up to 10 PM severity points. In groups “−1” and “0”, PM severity was close to 0, but outbreaks were up to 45 and 60 PM severity points, respectively. At the same time, in groups “−1” and “0”, mean PM severity was higher, at 2.42 and 2.22 points, respectively. In the group “+1”, PM severity was 7.86 in 2020, with a mean value of 4.29. Accessions from the group “+2” with the highest predicted positive effect on disease severity demonstrated average PM severity points of 30.00 and 38.75 in 2020 and 2022, respectively, with a mean value for the two years of 34.38. Detailed information on haplotype and PM severity for each accession is provided in Table S1. The best PM resistance (0 points in both years of observations) was found for haplotypes “CAGCAAA”, “CCGCAAG”, and “CAGCAGG”. The highest PM severity was observed for the haplotype “GCCAAGA” (35–60 points).

## 3. Discussion

### 3.1. PM Resistance in the Field and Its Relations with Yield Components

Environmental conditions greatly influenced the identification of QTLs for PM resistance, as the collection was unaffected by the disease in 2021. In that year, the temperature during the period from tillering to heading, when PM development would typically occur, was 2 °C higher than in 2020, and 4 °C higher than in 2022 (Figure 1). The amount of precipitation during the same period in 2021 was only 14.3 mm, which was dramatically lower than in 2020 (161.1 mm) and in 2022 (141.2 mm) (Figure 1). Together, higher temperature and lower precipitation in 2021 affected the development of *Bgh* and led to the absence of PM infection in the field. The considerable influence of weather conditions on PM development in the field is generally well-known [58], and this was further confirmed by our observations. As for 2020 and 2022, PM infection peaked at the end of booting and at the beginning of the heading stages. Most of the barley collection was resistant to PM in both years, although a broad spectrum of plant reactions was demonstrated (Figure S1A,B). Since most of the barley cultivars commercially grown in Kazakhstan are susceptible to PM [28], evolutionary changes of *Bgh* may result in the emergence and distribution of new pathotypes within the pathogen population. The present study provides new molecular tools for marker-assisted selection (MAS) of PM-resistant germplasm.

One of the most difficult main challenges facing breeders is developing cultivars that combine high yields with resistance to abiotic and biotic environmental factors. Relationships between PM severity and barley grain yields are controversial. Some studies have reported a negative effect of PM severity on yield components [59]. Another study described how the QTL decreasing yield in barley is linked to the PM resistance gene *Mlo* [60]. In our study, increased PM severity was associated with decreased NKS (Figure S1C) and increased TKW (Figure S1C,D). NKS reduction due to severe PM infection has been widely reported and is associated with a shortfall in photosynthesis post-anthesis because of damaged leaf surfaces [59]. At the same time, a reduction in gain number may increase their weight [61]. Thus, our experiment confirms the negative impact of PM severity on NKS with increasing TKW.

### 3.2. QTLs for PM Resistance

This study detected seven QTLs for PM resistance on three barley chromosomes, including three QTLs on chromosome 4H, three on chromosome 5H, and one on chromosome 7H (Table 1). QTLs identified on the same chromosome had been checked for LD (Figure 3). Results showed a low level of linkage among QTLs on chromosomes 4H and 5H: *R^2^* < 0.2, *p* < 0.0001; and *R^2^* < 0.1, *p* < 0.05, respectively (Figure 3). The only exception was QTL pair *Qhv_PM-4H.1*/*Qhv_PM-4H.3* (*R^2^* = 0.52, *p* < 0.0001) (Figure 3). Genetic positions of all QTLs were compared with known PM resistance genes and loci from the literature. The first QTL *Qhv_PM-4H.1* was located 1.5 Mbp away from another barley PM resistance QTL *Bgh-qtl-4H-11_10319*, which has been previously described in the literature [56]. The remaining two QTLs identified on chromosome 4H were *Qhv_PM-4H.2* and *Qhv_PM-4H.3*. A search of the literature showed that there are no PM resistance loci near these QTLs. Among PM-resistant genes, the most effective one is *mlo*, which was previously mapped on the long arm of chromosome 4H [30] and confers complete and broad-spectrum resistance against *Bgh*. However, this gene is located in the interval 589,324,720–589,327,859 bp [62], which is 77 Mbp away from the nearest QTL identified on chromosome 4H in the current study (Table 1). On chromosome 5H, QTL *Qhv_PM-5H.1* was identified as 2.5 Mbp away from PM resistance QTL *Qrbg_5H_1* [53] (Table 1). The other two QTLs, *Qhv_PM-5H.2* and *Qhv_PM-5H.3*, were located distantly from known PM resistance genes and QTLs. The only genetic factor described for PM resistance on this chromosome is *Mlj* [24]. However, no physical position was reported for that gene; therefore, it is uncertain if the two other identified QTLs on chromosome 5H, *Qhv_PM-5H.2* and *Qhv_PM-5H.3*, are associated with *Mlj*. The comparative assessment of *Qhv_PM-7H.1* on chromosome 7H (Table 1) suggests that it is 75 Mbp away from a gene encoding for MLO-like protein [62]. Thus, QTLs *Qhv_PM-4H.1* and *Qhv_PM-5H.1* demonstrated high significance and PVE, and a strong effect on PM severity, along with similar candidate loci found in the literature (Table 1). These findings suggest that these genomic regions play an important role in broad PM resistance in barley. As for the remaining five QTLs, based on the available data, to the best of our knowledge, no corresponding QTLs or genes have been described; hence, these QTLs can be considered as putatively novel factors for PM resistance. 

### 3.3. Promising Haplotypes for PM Resistance

Among the seven QTLs identified in this study, the minor alleles of four QTLs demonstrated a negative effect on PM severity in GWAS, and the minor alleles of three QTLs demonstrated a positive effect (Table 1). Depending on the allele status of the seven QTLs, 31 haplotypes were found in the studied barley collection (Figure 4A). According to GWAS results, in 21 of these, alleles with a negative effect on PM severity prevailed (haplotype groups “−3”, “−2”, and “−1”); five haplotypes had a neutral effect (group “0”), and the remaining five haplotypes had a positive effect on PM severity (haplotype groups “+1” and “+2”) (Table 1, Figure 4A). The growth of PM severity from haplotype group “−3” to group “+2” (Figure 4B) suggests the effectiveness of QTLs identified in the current study and provides information about promising accessions for the breeding of PM-resistant barley cultivars. For example, all accessions with haplotypes “CAGCAAA”, “CCGCAAG”, and “CAGCAGG” from group “−3” demonstrated 0 PM severity (Table S1) and, therefore, total resistance to *Bgh*. All three haplotypes had effective allele “C” of the QTL *Qhv_PM-4H.1*. This QTL possessed the largest PVE and produced the largest negative effect on PM severity (Table 1). At the same time, accessions with the haplotype “GCCAAGA” from group “+2” were susceptible to *Bgh*, and the severity of PM was high (Table S1). This haplotype carried alleles “A” and “G” of QTLs *Qhv_PM-5H.1* and *Qhv_PM-5H.3*, respectively, with moderate PVE and a positive effect on PM severity (Table 1). Generally, haplotype analysis confirmed GWAS results and allowed us to find promising haplotypes for the breeding and MAS of PM-resistant barley. QTLs, especially *Qhv_PM-4H.1*, *Qhv_PM-5H.1*, and *Qhv_PM-5H.3*, could be used for further analysis and trait pyramiding.

## 4. Materials and Methods

### 4.1. Barley Germplasm Panel and SNP Genotyping

A germplasm collection of 406 two-rowed spring barley accessions from the USA (*n* = 264), Kazakhstan (*n* = 95), Europe (*n* = 37), and Africa (*n* = 10) was used (Table S2) [57]. This collection was previously used for GWAS of barley grain quality traits [57]. Accessions from the USA and Kazakhstan were also used for GWAS of yield-related traits [63]. The American part of the collection and the SNP-genotyping data were obtained from the US Barley Coordinated Agricultural Project (CAP) [64,65]. The National Bioresource Project of Japan provided seed material and genotyping data of European and African accessions. Barley accessions from Kazakhstan were genotyped using the Illumina GoldenGate 9K SNP chip of the TraitGenetics company (TraitGenetics GmbH, Gatersleben, Germany). The results of SNP genotyping of accessions from Kazakhstan, USA, Europe, and Africa were merged and filtered by the minor allele frequency (MAF) > 0.05 and SNP with missing data < 0.1. Markers not meeting these requirements were removed from the analysis. In total, 1648 SNPs met all criteria and were selected for population structure analysis and GWAS. The SNP positions according to the Illumina iSelect2013 (cM) and Barley 50k iSelect SNP Array (bp) [66] map sets were obtained from the Triticeae toolbox database [67].

### 4.2. Field Trials and PM Resistance Evaluation

Field trials were conducted in three successive years (2020, 2021, and 2022) in the fields of the KRIAPG (43°14′03″ N and 76°42′00″ E, altitude 786 m). Barley accessions were sown using a nearest-neighbor randomized complete block design (nn-RCBD) with randomly assigned barley accessions. Each accession was grown in individual 1 m^2^ plots (15 cm spaces between neighboring plots) in two replications under rainfed conditions. The experimental design remained unchanged throughout the three-year period. Because PM is widespread in southeastern Kazakhstan, field trials relied on natural inoculation. The disease assessment was conducted twice: first, at the early booting stage (41–45 on the Zadoks scale); and second, during ear emergence (51–59 on the Zadoks scale) [68]. PM severity was assessed using a rating scale from 0 to 100 depending on the percentage of leaf and stem area infected, so that 0 represented the absence of infection and 100 indicated high susceptibility. The highest PM severity of each accession from the two replications of the two assessments per year was chosen for the GWAS. The data from 2021 were removed from the analysis due to a serious drought and the consequent absence of PM symptoms in the barley collection for that year.

In order to study relationships of PM severity with important yield-related traits, barley collection was assessed in terms of the number of kernels per spike (NKS, pcs), thousand kernel weight (TKW, g), and grain yield per m^2^ (YM2, g/m^2^). Pearson correlation testing was performed using the “corrplot” package for R [69]. IBM SPSS Statistics 2022 [70] was used for haplotypes boxplot construction.

### 4.3. Genome-Wide Association Study (GWAS), Linkage Disequilibrium (LD), Population Structure, and Haplotype Analysis

The GWAS was performed using the GAPIT v3 package [71] for R 4.0.2 and multi-locus mixed linear model (MLMM) [72]. For the assessment of the effect of population structure on GWAS results, the kinship matrix (K-matrix) was calculated in GAPIT with the Van Raden method. Population structure of the current barley collection was previously described using principal component analysis (PCA), neighbor-joining (NJ) tree, and Bayesian clustering in STRUCTURE [57]. The ancestry coefficient data (Q-matrix) were obtained from STRUCTURE. *p*-value < 3.14 × 10^−5^ (Bonferroni correction) and false discovery rate (FDR) < 0.05 were chosen as thresholds for significant associations. Linkage disequilibrium (LD) of marker pairs was calculated and plotted using TASSEL v5.2.84 [73]. For the haplotype analysis, the studied barley collection was divided into groups according to the sum of alleles of significant marker–trait associations (MTAs). Each allele in the haplotype was designated as −1 if its effect on the trait was negative, 0 if it was a non-effective allele, and +1 if the allele had a positive effect.

## 5. Conclusions

In the present study, a barley collection, including 406 accessions from the USA, Kazakhstan, Europe, and Africa, was screened for PM resistance under the field conditions of southeastern Kazakhstan over a three-year period. Phenotypic analysis showed sufficient variation in the PM resistance level over two years, and a negative effect of PM severity on the yield-related trait NKS. GWAS identified seven QTLs associated with PM resistance. Five of these QTLs are putatively new PM resistance factors. The remaining two QTLs had candidate QTLs from the literature, high significance and PVE, and strong effects on PM severity. Seven identified QTLs may be used for further analysis, trait pyramiding, and MAS. Additional haplotype analysis revealed three haplotypes with allele combinations demonstrating stable and complete resistance to PM and one haplotype associated with high PM severity. A germplasm with these haplotypes may be used as a donor for the breeding of PM-resistant barley cultivars.

## Figures and Tables

**Table 1:** Genome-wide association study (GWAS) results for two years of the experiment.

QTL	Marker	Chr.	Position (bp)	MAF	2020					2022					Candidate Loci
					*p*-Value	FDR *p*-Value	PVE (%)	Alleles	Effect	*p*-Value	FDR *p*-Value	PVE (%)	Alleles	Effect	
*Qhv_PM-4H.1*	12_20274	4H	3,623,098	0.139	1.2 × 10^−8^	0.000010	85.3	G/C	−6.3						*Bgh-qtl-4H-11_10319* [56]
*Qhv_PM-4H.2*	11_10509	4H	496,024,940	0.066	7.5 × 10^−6^	0.003696	0.0	C/A	−4.4						
*Qhv_PM-4H.3*	11_10914	4H	511,791,369	0.178	8.9 × 10^−6^	0.003696	0.0	C/G	−4.1						
*Qhv_PM-5H.1*	12_30980	5H	2,540,132	0.071						2.4 × 10^−8^	0.00004	20.6	C/A	5.4	*Qrbg_5H_1* [53]
*Qhv_PM-5H.2*	11_11240	5H	354,892,901	0.101	6.2 × 10^−10^	0.000001	0.0	A/T	5.0						
*Qhv_PM-5H.3*	12_10769	5H	646,543,760	0.051						2.6 × 10^−5^	0.02137	12.8	A/G	4.2	
*Qhv_PM-7H.1*	12_31513	7H	475,182,581	0.112	2.2 × 10^−5^	0.007203	0.4	A/G	−4.9						

Chr.—chromosome; MAF—minor allele frequency; FDR—false discovery rate; PVE—phenotypic variation explained.

## Data Availability

The datasets generated and/or analyzed during the current study are available in the manuscript text and/or Supplementary Materials.

## Supplementary Materials

The following supporting information can be downloaded at: https://www.mdpi.com/article/10.3390/plants12122375/s1, Figure S1: Phenotypic diversity of PM resistance in studied barley collection in (A) 2020 and (B) 2022. Pearson correlation coefficients € between PM severity and yield-related traits in (C) 2020, (D) 2022, a€ (E) mean values. Figure S2: Population structure in studied barley collection. (A) Principal component analysis (PCA) plot, (B) neighbor-joining (NJ) tree, and (C) Bayesian clustering of the 406 barley accessions for *K* from 2 to 5. Table S1: Haplotypes and PM severity scores for each accession of studied barley collection. Table S2: The list of barley accessions used in the study.
