# Supplementary material for: Identification of Quantitative Trait Loci Associated with Powdery Mildew Resistance in Spring Barley under Conditions of Southeastern Kazakhstan
Source: Plants (Basel). 2023 Jun 19;12(12):2375. doi: 10.3390/plants12122375 (PMC10301996; doi:10.3390/plants12122375)
Supplement: Supplementary file 1 [file plants-12-02375-s001.zip › Supplementary Materials Figures S1 and S2.pdf]

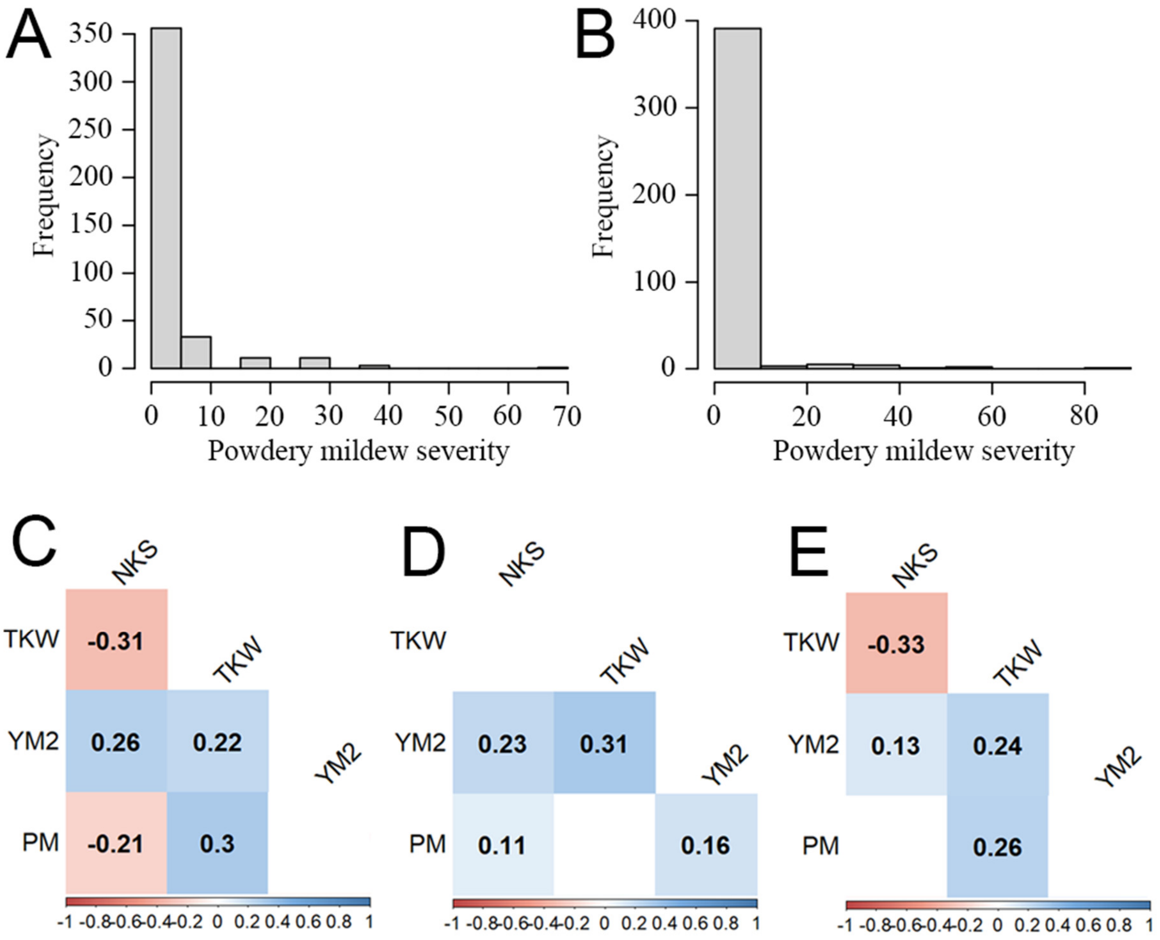

Figure S1. Phenotypic diversity of PM resistance in studied barley collection in (A) 2020 and (B) 2022. Pearson correlation coefficients € between PM severity and yield-related traits in (C) 2020, (D) 2022, a€ (E) mean values.

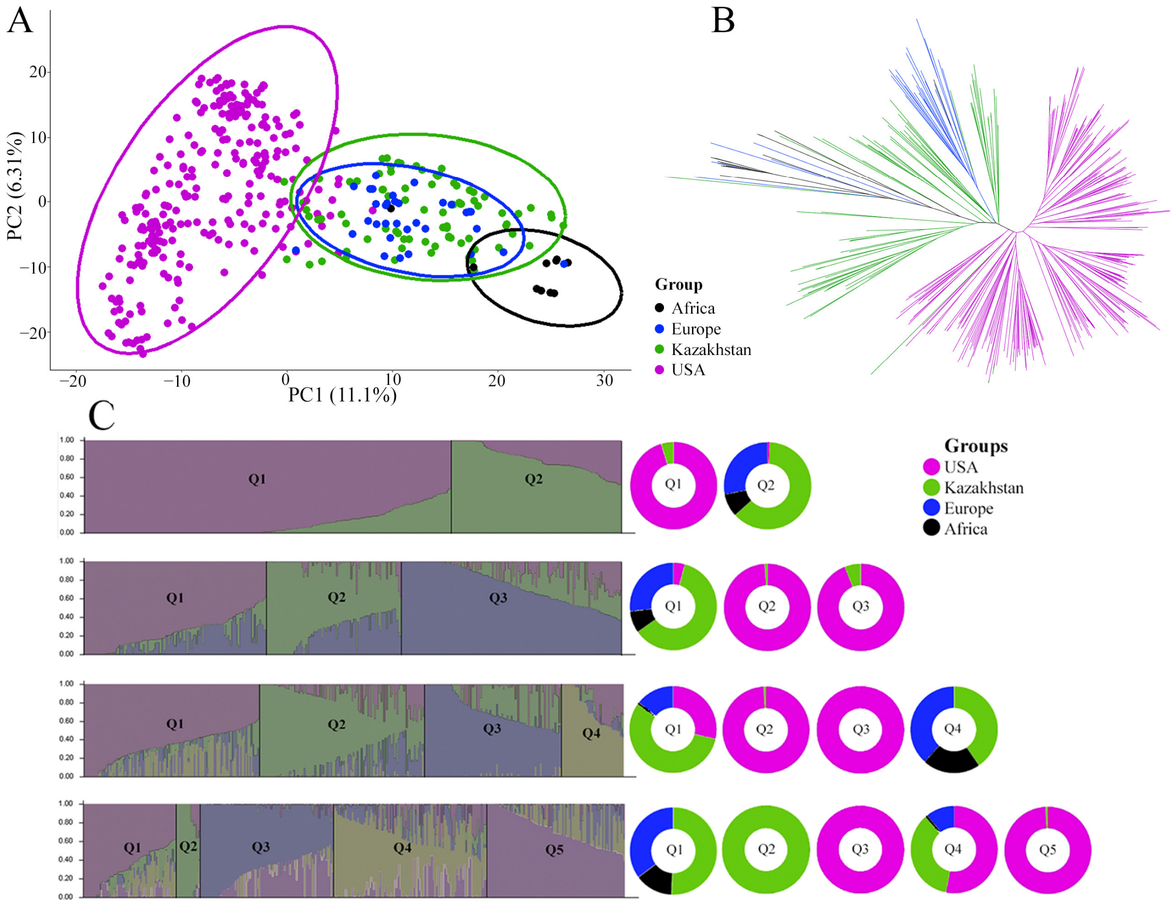

Figure S2. Population structure in studied barley collection. (A) Principal component analysis (PCA) plot, (B) neighbor-joining (NJ) tree, and (C) Bayesian clustering of the 406 barley accessions for *K* from 2 to 5.
